# Menopausal Symptoms and Its Correlates: A Study on Tribe and Caste Population of East India

**DOI:** 10.1155/2015/984767

**Published:** 2015-07-30

**Authors:** Doyel Dasgupta, Priyanka Karar, Subha Ray, Nandini Ganguly

**Affiliations:** Department of Anthropology, University of Calcutta, 35 Ballygunge Circular Road, Kolkata, West Bengal 700019, India

## Abstract

Present study aimed to compare the incidence of menopausal problems and concomitants between tribe and caste population. This cross section study was conducted in five villages of West Bengal, a state in the eastern part of India. This study was conducted between two different ethnic groups—one of the “Particularly Vulnerable Tribal Groups (PTG)” of India named as “Lodha” and the other was a Bengali speaking caste population. A total number of 313 participants were finally recruited for this study. Study participants were married, had at least one child, had no major gynaecological problems, and had stopped menstrual bleeding spontaneously for at least 1 year. Additionally, data on sociodemographic status and menstrual and reproductive history were collected using a pretested questionnaire/schedule. Bivariate analyses (chi square test) revealed that significantly more number of caste participants suffered from urinary problems than their tribe counterpart. The reverse trend has been noticed for the frequency of vaginal problems. Multivariate analyses (binary logistic regression) show that sociodemographic variables and menstrual and reproductive history of the present study participants seem to be the concomitants of menopausal symptoms. Tribe and caste study population significantly differed with respect to the estrogen deficient menopausal problems and the concomitants to these problems.

## 1. Introduction

Menopause is associated with reduction in the normal estrogen levels and subsequent incidence of menopausal symptoms [1]. Studies stated that, during the early postmenopausal period, the prevalence of vasomotor symptoms ranges from 30 to 80 percent and, during later period, vaginal dryness from 25 to 47 percent [2]. The prevalence of urogenital complaints has been reported to increase at menopause and is more common in women than men, implicating menopause [3–5].

For most women, natural menopause takes place between the ages of 45 and 55 years [6, 7]. In most developed countries, this event occurs around the age of 50 years [8]. But in developing countries like India there has been a trend in advancement of age at menopause [9]. Thus, along with increase in life expectancy growing number of these women can expect to live for several decades after menopause. Furthermore, several studies have shown that the frequency of reporting of menopausal symptoms of Indian women varies with culture and also with sociodemographic status [10, 11].

In Indian subcontinent, one may find an appreciable quantum of literature on menopause. To the best of the understanding of the authors, few studies addressed the menopausal issues of any indigenous ethnic group (tribe). Although in this globalised world we observe some radical change to have occurred in different societies, the indigenous ethnic group maintains homogeneity in the form of practicing endogamy and in maintaining traditional cultural beliefs. They differ from the other ethnic groups in terms of their biological and cultural identity such as mating structure and social tradition. This inequality in social and cultural characteristics is likely to affect the reproductive characteristics, including reproductive aging or menopause of the indigenous groups differentially.

Under this circumstance, the present study aims to find out the prevalence of menopausal problems between tribe and caste population of West Bengal, a state of east India.

## 2. Material and Methods

### 2.1. Study Area

The present study has been conducted in one of the indigenous ethnic groups known as “Lodha” of West Bengal, a state located in eastern India. Lodha population have been declared as one of the “Particularly Vulnerable Tribal Groups” of this country on the basis of certain characteristics like low level of literacy, preagricultural level of technology, and declining or stagnant population. In West Bengal they mainly lived in the district of West Medinipur. We selected them from 5 villages of West Medinipur district. Caste population have been recruited from the same villages. Study areas have been selected using multistage random sampling techniques, considering one local administrative unit (*gram panchayat*) from the district and five villages from* gram panchayat*. Each of the administrative units was randomly selected.

### 2.2. Study Population

We identified 404 postmenopausal (221 Lodha and 183 caste participants) participants on the basis of the criteria fixed for the study: the participants were between the ages of 40 and 55 years, had attained natural menopause, were married, had at least one child, and had no history of hysterectomy or other major gynaecological problems. We have excluded childless participants from the study to ensure that all participants have been exposed to certain reproductive events/behaviour in life (like pregnancy, lactation, and parity) that modify the reproductive hormonal levels of participants. Finally 313 of these participants (172 Lodha and 141 caste participants) were available or volunteered to participate. Thus the total participation rate was 77.5.9% (lodha 77.8% and schedule caste 77.0%). The menopausal state was ascertained following the classification of the World Health Organization [8].

Prior to collection of data, the nature of research was explained and consent was taken from all the participants.

### 2.3. Data Types and Data Collection Techniques

A pretested and structured questionnaire was used to collect the data on sociodemographic status that include age of the participants at the time of interview, years of education, working status, per capita monthly household expenditure in Indian rupees, use of tobacco, and consumption of black tea or alcohol. Data on menstrual history includes ages at menarche, history of menstrual regularity, and history of heavy and scanty menstrual discharge; reproductive history includes age at marriage, use of oral contraceptive pills, number of live births, duration of breastfeeding (months), and history of sterilization. Barring the variable “age at menarche,” information on other menstrual and reproductive factors pertains to the stage after the last child birth. Data collection techniques have been discussed elsewhere [12]. Menopausal problems of the participants were collected (last 30 days' recall) with the help of a “menopausal problem list” used in study on the Bengali speaking Hindu women of West Bengal [13]. The procedural details of using this questionnaire have been discussed elsewhere [13].

The present study is conducted during the period of March 2009 to July 2012. The study protocol has been approved by the “Research Ethics Committee” of the University of Calcutta, India (reference number: BEHR/1095/2304).

### 2.4. Methods of Analyses

We applied descriptive statistics (frequency and mean calculation) to compare the trend in the sociodemographic variables and menstrual and reproductive history and menopausal problems between tribe and caste postmenopausal participants. We also used *t*-test and *χ* ^2^ test in bivariate comparisons.

Binary multivariate logistic regression (using enter method) analyses were done to find out the concomitants that significantly associated with menopausal problems. In these analyses, the presence or absence of menopausal problems were considered dependent variables. All the sociodemographic, menstrual, and reproductive variables were entered together in the analysis as independent variables. The following were the reference categories (in parenthesis) for each of the categorical variables: working status (nonworking), history of heavy or scanty menstrual discharge (absent), oral contraceptive pill (ever used), history of reproductive wastage (absent), sterilisation (absent), use of tobacco (no), and consumption of black tea (no). The rest of the variables, such as age of the participants at the time of interview, per capita monthly household expenditure, years of education, duration of breast feeding, and number of live births, were treated as continuous variables. The colinearity of the independent variables is checked and the values are found to be within the acceptable limit (SE = 0.001 to 5.0). In the text, values of odds ratio and 95% CI are presented only for those variables that showed significant association (*P* ≤ 0.05) with a particular menopausal problem. We excluded the variables like alcohol consumption from the multivariate analyses as none of the caste population and few sections of tribal women consume it.

## 3. Results

Table 1 shows that an overwhelming section of tribal participants had no formal education whereas more than half of the caste women got it. Larger section of caste participants belonged to nonworking category, but higher number of tribal participants were mostly engaged in day labour, small scale business, and agricultural activities. Per capita monthly household expenditure was significantly more in caste than in tribal participants. Significantly higher number of tribal participants were tobacco chewers and majority of participants consumed black tea. A few sections of tribal participants consume alcohol, but none of the caste ones consumes it. No significant differences have been observed between tribe and caste participants regarding mean age at menarche. An overwhelming section of participants had the history of menstrual regularity, whereas significantly higher number of tribal participants had the history of heavy and scanty menstrual discharge. Mean ages at marriage, first pregnancy, and last pregnancy and duration of breastfeeding were significantly earlier in tribal than caste participants. Mean number of live births was similar among both groups. Use of oral contraceptive and history of reproductive wastage was significantly higher in caste than tribe participants, whereas significantly higher number of tribal participants have the history of sterilization. Tribal participants attained menopause at an earlier age than caste women.

Table 2 shows the frequency of menopausal symptoms of tribe and caste participants. Vasomotor problems like hot flush and night sweats did not differ significantly between tribe and caste participants. Urinary problems like inability to hold urine and urine leakage were significantly higher in caste participants than intribal ones. On the other hand, vaginal problems such as vaginal dryness, discharge, and bad smell were significantly higher among tribal than caste participants.

Table 3 shows that the chance of hot flush in tribal and caste participants increased with early age at menarche and early age at last pregnancy, respectively. The history of reproductive wastage increased the chance of night sweat among tribal participants. On the other hand the chance of this problem significantly increased in caste women with early onset of menarche, with history of scanty menstrual bleeding, and never use of oral contraceptive pills. Among the tribe, history of heavy menstrual discharge significantly increased the likelihood of the problem like painful urination but, among caste, late onset of menarche and age at marriage seemed to be the factors associated with this problem. Tribal participants who consume black tea and caste ones who use the oral contraceptive pills were more likely to be affected by inability to hold urine and frequent urination. Present age was a concomitant for urine leakage in tribal participants, whereas use of oral contraceptive pills and sterilization were found to be the concomitants for this problem for caste women. Presence of heavy and scanty type of menstrual discharge increased the chance of vaginal dryness in tribe and caste participants, respectively. In tribal participants present age, history of heavy menstrual discharge, and short duration of breastfeeding significantly increased the chance of vaginal discharge whereas present age was the only factor for caste participants. Concomitants like short duration of breastfeeding increased the chance of burning sensation of tribe; on the other hand fewer years of education and presence of scanty menstrual discharge significantly increased the likelihood of this problem in caste participants. Decrease in per capita monthly household expenditure and history of scanty menstrual discharge increased the chance of vaginal itching in tribe and caste participants, respectively, whereas decreased duration of breastfeeding and decreased number of live births significantly increased the likelihood of bad smell in vagina in tribe and caste participants, respectively.

## 4. Discussion

Menopause may have profound implications for subsequent morbidity and mortality [14]. Studies show that women who attain menopause at an early age are at a greater risk of being affected by cardiovascular disease [15], osteoporosis [16], and rheumatoid arthritis [17], while late menopause carries a higher risk of breast [18] and endometrial [19] cancer. The estrogen deficient menopausal problems lead to an increase in the progression of cytokines such as GM-CSF, IL-1, and IL-6, and that could potentially induce autoimmune responses in systemic autoimmune diseases such as SLE and rheumatoid arthritis [17, 20]. Aging is also associated with progressive decline in T cell functions, including decreased response to various antigens, and production of IL-2, and defect in signalling pathway resulting in the increase in frequency of cancer [21, 22].

Studies stated the relationship between hot flashes and certain reproductive history variables, such as age at menarche, age at first and last pregnancy, and parity; however it revealed the inconsistent result [13, 23, 24]. However, the present study showed that the likelihood of hot flush was more in the participants who attained menarche at an early age and had last pregnancy at their early age. Early onset of menarche might be associated with early exhaustion of ova [25] and early age at last pregnancy might be related with faster rate of atresia [26]. The ovarian shortage of oocytes for both reproductive events could formulate the fluctuation of oestrogen level during menopausal transition and occurrence of hot flush [27].

Consumption of black tea seemed to be the factor of urinary incontinences (inability to hold urine and frequent urination) of tribe participants whereas, along with the use of oral contraceptive pills, lower incidence of sterilization seemed to be the factor of urinary incontinences of caste ones. In this context, it has been stated that the development of UI symptoms can also be affected by caffeine intake in the form of black tea. A study on women from USA reported that the incidence of UI was positively associated with caffeine ingestion, and a high level of intake increased the risk of urge but not stress or mixed incontinence [28]. Present study was corroborated with that finding. It has been found that caffeine has diuretic effect; thus intake of such substance can lead to rise in the pressure that is exerted inwards by the detrusor urinae muscles of the bladder wall, and this detrusor instability may produce urinary incontinence [29, 30]. Furthermore, earlier study stated that both the current and the former use of oral hormone therapy increased the risks of urinary incontinence (UI) among postmenopausal participants [31, 32]. Use of the hormone changed the collagen composition of the bladder and makes it contractile. Increased bladder contractility has been proposed as some potential explanations of the occurrence of UI [31, 32]. As OCP use was significantly more in caste study participants of this study compared to tribe ones, that might be a reason of more prevalence of urinary symptoms in caste participants.

Oestrogen is a dominant regulator of vaginal physiology. It has been found that oestrogen-receptor density is highest in the vagina [33–35]. Several features of the vaginal microenvironment change have been noticed with increasing age, mostly in response to alterations of oestrogen levels [35]. Present study was consistent with this finding. During menopause, the vaginal mucosa becomes weakened, loses its rugae, and appears pale and almost transparent because of decreased vascularity [36]. Present study showed that tribal study participants who had the history of heavy menstrual discharge and breastfeed their child for short time are more likely to be affected with the estrogen deficient symptoms like vaginal problems during her menopausal life. Whereas for a caste study participants, who had the history of scanty menstrual discharge and less number of live births, the chance of these types of problems increased. In this regard it has been said that short duration of breastfeeding and lower number of live births lead to the fast depletion of ovarian follicles [37]. As follicular decline results in lowered levels of oestrogen, faster exhaustion of ovarian follicle might be a reason of fluctuation of oestrogen that might be associated with occurrence of vaginal problems. Moreover, oestrogen deficiency was one of the reasons for heavy or scanty menstrual discharge [38]. So women who had the history of scanty menstrual discharge were more likely to suffer from vaginal symptoms during menopause [13].

Menopausal health of women is determined by their menstrual and reproductive histories, sociodemographic variable, and types of diet. They are susceptible to health problems by reason of either their genetics or their lifestyles and, finally, their access to adequate health care [13, 39, 40]. Thus, inclusion of the data on dietary practices and genetics would give a better understanding of menopausal symptoms of the two different populations of the present study. Moreover, the findings of the present study suggest that variation exists in the menopausal experience and its socio demographic and reproductive concomitants between two ethnic groups living in the same geographical area. However, they are limited in generalizability due to the small sample size. Our volunteer sample may not be representative of the broader population of postmenopausal women from each of the ethnic groups.

## Figures and Tables

**Table 1:** Socio demographic variables, menstrual and reproductive history.

	Tribe (*n* = 172)	Caste (*n* = 141)	*P* value
Mean age of participants at the time of interview (years)	49.09 ± 4.7	48.85 ± 4.2	0.052
Years of education			NA
0	163 (94.6)	66 (46.8)	
1–4	5 (3.0)	13 (9.2)	
5–8	3 (1.8)	37 (26.2)	
9-10	1 (0.6)	18 (12.8)	
≥11	0 (0.0)	7 (5.0)	
Working status			0.097
Nonworking	50 (29.1)	110 (78.0)	
Working	122 (70.9)	31 (22.0)	
Mean per capita monthly household expenditure (in Indian rupees)	998.35 ± 22.2	1080.96 ± 27.8	0.001
Use of tobacco (at least once in a week)	122 (70.9)	28 (19.9)	0.001
Consumption of black tea (at least once in a week)	145 (84.3)	120 (85.1)	0.844
Consumption of alcohol (at least once in a week)	13 (7.6)	0 (0.0)	NA
Mean age at menarche (years)	13.94 ± 1.4	14.10 ± 1.6	0.443
History of menstrual regularity	169 (98.3)	335 (98.2)	0.990
Had periods with heavy discharge	69 (40.1)	63 (18.5)	0.001
Had periods with scanty discharge	47 (27.3)	30 (8.8)	0.001
Mean age at marriage (years)	15.07 ± 2.5	17.29 ± 3.4	0.001
Mean age at first pregnancy (years)	18.23 ± 2.4	20.05 ± 3.1	0.001
Mean age at last pregnancy (years)	25.96 ± 5.4	27.21 ± 4.8	0.009
Mean number of live births	3.70 ± 1.77	3.75 ± 1.5	0.738
Mean duration of breastfeeding of the last child (months)	51.16 ± 20.8	37.81 ± 32.2	0.001
Ever use of oral contraceptive pills	1 (0.6)	15 (10.6)	0.001
History of reproductive wastage	12 (7.0)	29 (20.5)	0.004
History of sterilisation	121 (70.3)	85 (60.3)	0.040
Mean age at menopause (years)	41.69 ± 5.6	40.65 ± 5.0	

Figures in the parentheses indicate percentage.

**Table 2:** Prevalence of menopausal symptom types.

	Tribe	Caste	*P* value
Vasomotor domain			
Hot flush	66 (38.4)	57 (40.4)	0.400
Night sweats	94 (54.7)	76 (53.9)	0.492
Urinary domain			
Painful urination	44 (25.6)	25 (17.7)	0.062
Inability to hold urine	81 (47.1)	91 (64.5)	0.001
Frequent urination	50 (29.1)	54 (38.3)	0.054
Urine leakage	41 (23.8)	72 (51.1)	0.001
Vaginal domain			
Vaginal dryness	79 (45.9)	10 (7.1)	0.001
Burning sensation	16 (9.3)	7 (5.0)	0.105
Vaginal discharge	56 (32.6)	19 (13.5)	0.001
Vaginal itching	24 (14.0)	17 (12.1)	0.374
Bad smell	20 (11.6)	3 (2.1)	0.001

Figures in the parentheses indicate percentage.

**Table 3:** Correlates of menopausal symptoms.

	Tribe	Exp(*B*), 95% C.I., *P* value	Caste	Exp(*B*), 95% C.I., *P* value
Hot flush	Age at menarche (in years)	0.139 (0.027–0.719) 0.019	Age at last pregnancy (in years)	0.251 (0.091–0.692) 0.008

Night sweat	History of reproductive wastage (absent)	1.001 (1.000–1.003) 0.046	Age at menarche (in years)	0.916 (0.854–0.983) 0.015
			Scanty menstrual discharge (absent)	0.694 (0.511–0.944) 0.020
			Use of OCP (ever used)	1.678 (1.081–2.603) 0.021

Painful urination	Heavy menstrual discharge (absent)	1.894 (0.816–0.978) 0.015	Age at menarche (in years)	1.547 (1.058–2.264) 0.024
			Age at marriage (in years)	1.431 (1.019–2.009) 0.039

Inability to hold urine	Consumption of black tea (no)	3.029 (1.140–8.049) 0.026	Use of OCP (ever used)	0.738 (0.548–0.994) 0.046

Frequent urination	Consumption of black tea (no)	1.606 (1.041–2.477) 0.032	Use of OCP (ever used)	0.716 (0.526–0.974) 0.033

Urine leakage during laughing & coughing	Present age (years)	1.906 (0.829–0.989) 0.028	Use of OCP (ever used)	0.670 (0.499–0.900) 0.008
			History of sterilisation (absent)	1.288 (1.000–1.659) 0.050

Vaginal dryness	Heavy menstrual discharge (absent)	0.326 (0.141–0.754) 0.009	Scanty menstrual discharge (absent)	0.112 (1.161–3.840) 0.014

Vaginal discharge	Present age (years)	1.894 (0.816–0.978) 0.015	Present age (years)	1.029 (1.006–1.054) 0.015
	Heavy menstrual discharge (absent)	0.369 (0.146–0.934) 0.035		
	Duration of breastfeeding last child	0.130 (0.071–0.060) 0.013		

Burning sensation at vagina	Duration of breastfeeding last child	0.6977 (0.5283–0.9212) 0.014	Years of education	0.932 (0.881–0.987) 0.016
			Scanty menstrual discharge (absent)	2.391 (1.203–4.749) 0.013

Vaginal itching	Per capita monthly household expenditure (in Indian rupees)	0.396 (0.189–0.832) 0.014	Scanty menstrual discharge (absent)	0.827 (1.081–3.087) 0.024

Bad smell at vagina	Duration of breastfeeding last child	0.364 (0.207–0.639) 0.001	Number of live births	0.07 (0.11–0.25) 0.012

Only significant concomitants have been presented here.
